# Multi-stage surgery combined with radiotherapy for treatment of giant anterior chest wall keloid: A case report

**DOI:** 10.1097/MD.0000000000018886

**Published:** 2020-01-24

**Authors:** Qingwu Liu, Ping Li, Zhishan Yang, Baoquan Qu, Chunfang Qin, Shengnan Meng, Huijuan Fang, Ruiying Wu, Tiantian Cheng, Dingquan Yang

**Affiliations:** a School of Clinical Medicine, Beijing University of Chinese Medicine; b Beijing Institute of Traditional Chinese Medicine, Beijing Hospital of Traditional Chinese Medicine Affiliated to Capital Medical University; c China-Japan Friendship Hospital, Beijing, China.

**Keywords:** chest wall, giant keloid, multistage surgery

## Abstract

**Rationale:**

Giant keloids often have indications for surgical resection, but postoperative reconstruction of the skin and high recurrence of keloids are a challenge for clinical treatment. This article reports a rare successful treatment of a giant keloid in the anterior chest wall by multistage surgery combined with radiotherapy, which is why this case is meaningful.

**Patient concerns:**

A 66-year-old woman presented a giant keloid with ulcerations and severe itching on the anterior chest wall. She had a history of keloid disease for more than 10 years, and had been treated by multiple operations, with no success.

**Diagnoses:**

The patient was diagnosed as keloid based on her history and symptoms. Histopathology findings supported our diagnosis.

**Interventions:**

We successfully excised the keloid after 5 operations and 2 rounds of electron-beam radiotherapy, which was applied at 24 hours after the 4th and 5th operation.

**Outcomes:**

There was no sign of recurrence over the follow-up period of 24 months.

**Lessons:**

The combination of multistage surgery and radiotherapy presents as a good choice for the treatment of giant keloids.

## 1 Introduction

Keloids are benign dermal fibroproliferative tumors, characterized by massive proliferation of fibroblasts and excessive deposition of extracellular matrix.^[1]^ Keloids often appear following skin surgery or infection, but the etiology is still not completely understood.^[2]^ Due to pain, pruritus, aesthetics, and other dysfunctions caused by keloid contracture, keloid patients have a strong desire to seek treatment. There are many treatment modalities, including: surgery, intralesional corticosteroid injection, pressure therapy, radiation, cryotherapy, silicone gel application and laser therapy. These have been described to prevent the relapse of keloids. However, the efficacy of these methods is limited.^[3]^ The only technique that offers better results is radiotherapy, with success rates above 80%.^[2]^ Keloids tend to occur in areas with high tension, such as the anterior chest, back, neck, shoulders, arms. For female patients, keloids in the anterior chest are more likely to grow larger due to continuous pulling from the breasts.

Giant keloid resection is usually a major operation, and surgical techniques such as skin grafting and flap transfer are often required to close the wound after keloid removal. For elderly patients, it is difficult to grasp the pros and cons of disease treatment and adverse reactions/complications due to the general condition and risks of anesthesia. More seriously, flap grafting or the use of a skin dilator can also lead to the formation of skin/subcutaneous keloid tissue,^[4]^ making keloids even more difficult to treat. Here we report the successful treatment of a giant keloid in the anterior chest wall of an elderly woman by multi-stage surgery combined with radiotherapy.

## 2 Case report

**Figure 1:**
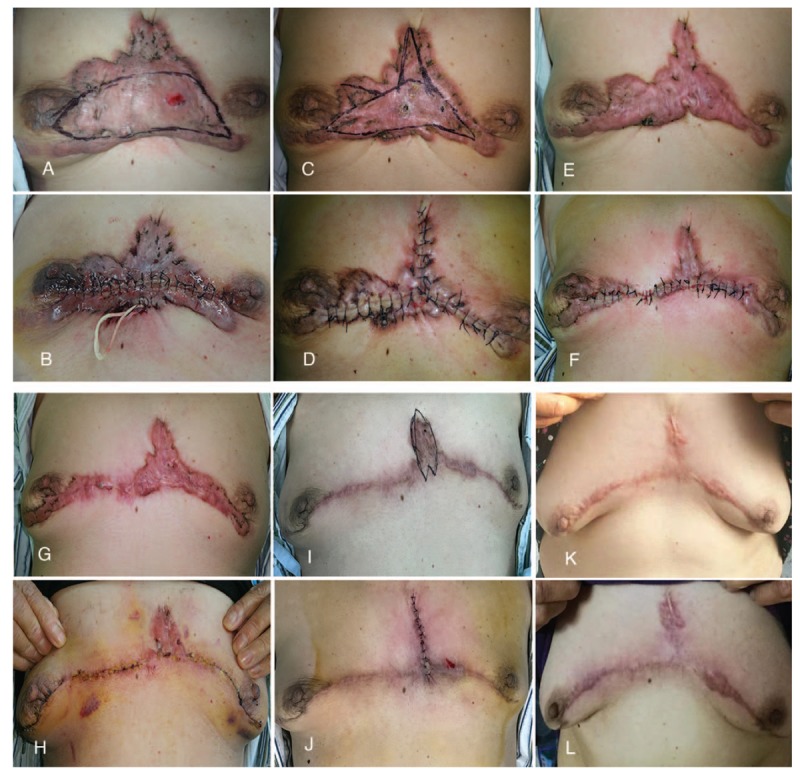
A, Giant keloid on the chest. B, First postoperative. C, Before the second operation. D, After the second operation. E, Before the third operation. F, After the third operation. G, Before the fourth operation. H, After the fourth operation. I, Before the fifth operation. J, After the fifth operation. K, 1 year after the multistage surgery. L, 2 year after the multistage surgery.

A 66-year-old woman was diagnosed with a recurrent giant anterior chest wall keloid, which she had had for more than 10 years. Past history consisted of repeated failed excisions and intralesional steroid injections. The patient suffered from local itching and pain, as well as progressive enlargement of the keloid. What was worse was that due to the giant keloid pulling the breasts on both sides of the chest skin, the patient could not walk upright. She felt pain and itching from the keloid and her breathing was also affected, resulting in poor sleep. On physical examination, a large 17 cm (L) × 8 cm (W) × 2 cm (T) epidermal mass was seen on the midline of the chest in the middle of both breasts, with an ulceration and small amount of exudation (Fig. 1). The patient had a history of hypertension.

**Figure 2:**
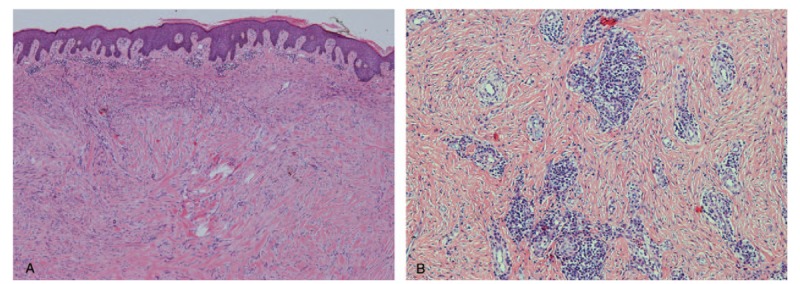
A, A large number of coarse and uniform eosinophilic red-stained collagen bundles are visible in the dermis, which are disorderly arranged. The collagen bundles are rich in mucin. The fibroblasts are active in the periphery of the lesions, the central distribution is reduced, the skin appendages are atrophied, and the vascular hyperplasia is in the dermis [hematoxylin-eosin (H&E), 40]. B, Inflammatory cell infiltration is observed around the blood vessels [H&E, 100].

We considered that the patient had not been treated with adjuvant therapy (e.g., radiation or injection therapy) after the initial surgery, and that the irregular follow-ups after surgery was one of the reasons for the increase in her keloid size. In addition, the hyperplasia of keloids destroys the hair follicles and sebaceous gland structure of the local skin, which results in the sebum being blocked. As well, the itching caused by keloid hyperplasia prompts the patient to scratch her skin, resulting in repeated infection and ulceration of the local keloid tissue. The new wound of skin stimulates the growth of the keloid, which aggravates the disease. Taking the patient's age and the risk of hypertension into account, we did not choose a one-time resection of the keloid, but instead a treatment of multistage surgery combined with radiotherapy. Histological analysis revealed typical keloid features (Fig. 2).

### 2.1 Surgical step

Before each operation, the resection area was evaluated. After the single resection, the resection area was directly sutured and the keloid was removed as much as possible. Subcutaneous and intradermal reduction sutures were used for absorbable sutures, and the leather surface was sutured with non-absorbable lines. The 5 mg of the betamethasone injection was injected locally around the wound immediately after the operation. The wound was covered with sterile gauze and pressurized with an elastic bandage.

### 2.2 Multistage surgery

On December 2nd of 2014, we designed a lateral fusiform incision to remove the keloid, of which about 15.0 cm (L) × 4.0 cm (W) × 2.0 cm (T) in size was removed (Fig. 1A, B). Due to the large radiation area after surgery, radiologists did not recommend radiation therapy because of the risk of radiation carcinogenesis.

On May 12th of 2015, before the second operation, the color of the keloid became lighter. We designed a lateral fusiform and a longitudinal incision, removing the keloid, of which about 15.0 cm (L) × 3.0 cm (W) × 1.0 cm (T) in size was removed (Fig. 1C, D). This relieved the tension between the two breasts, and improved the patient's thoracic activity.

On December 15th of 2015, before the third operation, the color of the keloid was obviously rosy and the patient reported relief from pain and itching. We designed a lateral fusiform incision to remove the keloid, of which about 15.0 cm (L) × 2.0 cm (W) × 1.0 cm (T) in size was removed (Fig. 1 E, F).

On May 31st of 2016, at the time of the fourth operation, the distance between the breasts on both sides was almost normal, and the thoracic activity of the patient returned to normal. We extended the length of the incision and remove the previously uncut keloid located under the breasts, of which about 20.0 cm (L) × 1.0 cm (W) × 1.0 cm (T) in size was cutted off (Fig. 1 G, H). Radiotherapy was applied within 24 hours after surgery at a dose of 15 Gy (3 Gy/day × 5 days).^[5]^

On March 9th of 2017, we designed the longitudinal fusiform incision for the fifth operation, and removed the keloid in the longitudinal direction, of which about 5.0 cm (L) × 2.0 cm (W) × 0.5 cm (T) in size was removed (Fig. 1 I, J). Radiotherapy was performed after the operation, and the dose was the same as before.

During the first to third interoperative period, patient was treated once every 4 to 8 weeks with intralesion injections of 5 mg of betamethasone and 2 mg of pingyangmycin. After the third operation, the scar volume was significantly reduced and showed no ulceration and exudation. The intraoperative injection treatment was changed to topical halomethasone cream, imiquimod cream, and asiaticoside cream.

At 1 year after surgery, mild hyperplasia was found in the wound, and local injections of betamethasone and 5-fluorouracil were given (Fig. 1K). At 2 years after surgery, no significant recurrence was observed (Fig. 1L). The patient was highly satisfied with her result.

## 3 Discussion

Surgery is the most direct method for treating keloids, but the recurrence rate of single surgery is extremely high, at 50% to 100%.^[6]^ The multistage surgery is where multiple resections are performed on a giant lesion, to finally completely remove the lesion. Due to the absence of skin dilators and the risk of secondary scarring caused by skin grafting and flaps, the multistage surgery simplifies the surgical procedure for giant keloids.

The principle is to use the elasticity of the skin itself to make the skin grow and stretch over time, and to remove the scar as much as possible under conditions where the incision can be directly sutured. After surgery, keloids tend to change from round or irregular to long strips. After the last resection, the wound was linear.^[7,8]^ The time of the next operation is determined according to the different surgical sites and the tension of the surgical incision. In general, surgery should be separated by about half a year to give the skin sufficient time to grow. The wounds and tension formed by multistage surgery are smaller, and the scar can also be treated during the no-operation periods to reduce the risk of recurrence. It is also very important to perform pathological examination of the removed keloids after surgery to exclude the possibility of malignant transformation.

Due to the histology of keloids, especially at the margin of the scar tissue, the keloid morphology is wider than its appearance.^[9,10]^ According to the recommendation of Jiao et al,^[10]^ the surgical resection range should be slightly wider than the keloid by 0.3 ∼ 0.5 cm. Subcutaneous separation of the incision edge can reduce the tension of the incision, which is conducive to the healing of the incision. After separation, single PDS-II absorbable suture (3–0) is used subcutaneously and single PDS-II absorbable suture (4–0) is used intracutaneously. The PDS-II absorbable wire maintains the low tension of the incision for 90 days. The skin was sutured with a single nylon thread. The removal of the leather suture line within 7 days can alleviate the local irritation reaction, and prevent the “squatting” phenomenon of the formation of the suture indentation.

At present, there is no single preferred treatment for keloids.^[11]^ Therefore, adjuvant therapy is recommended after surgery to prevent the recurrence of keloids. Surgery combined with postoperative radiotherapy can reduce the recurrence rate of keloid after surgery by 10% to 20%.^[12]^ However, the effects of surgery combined with radiotherapy for keloids is closely related to both the method of surgery and the timing and dose of radiotherapy. The Chinese keloid clinical treatment recommendation guidelines advocate radiotherapy within 24 hours after surgery,^[13]^ as immediate postoperative radiotherapy can reduce fibroblasts proliferation and reduce collagen fiber synthesis. As for the drugs the patient received during the operation period, both betamethasone and halomethasone are long-acting glucocorticoids that are anti-inflammatory, inhibit fibroblasts proliferation and collagen synthesis, and reduce collagen deposition.^[14]^ Pingyangmycin is an anti-tumor antibiotic found for the first time in Pingyang County, Zhejiang Province, China.^[15]^ China approved the clinical use of pingyangmycin in 1978 by the Chinese Food and Drug Administration (SFDA).^[16]^ Its main active component is bleomycin A5, which is a member of the bleomycin (BLM) family. Pingyangmycin induces G2/M cell cycle arrest in cancer cell lines, and induces apoptosis,^[17]^ down-regulates EGFR expression in tumor cells,^[18]^ induces apoptosis of vascular endothelial cells,^[19]^ and shares the same cytotoxic pathway with bleomycin.^[17]^ In China, Pingyangmycin has been widely used in the treatment of keloids,^[20]^ vascular malformations,^[21]^ warts^[22]^ and so on. Imiquimod 5% cream can regulate immunity and enhance local production of cytokines such as interleukins, tumor necrosis factor and interferons.^[23]^ It is currently used for HPV related anogenital disease,^[24]^ and superficial basal cell carcinoma.^[25]^ Imiquimod can down-regulate the production of TGF-β by stimulating interferon production.^[26]^ In addition, imiquimod has an antiangiogenic effect, thereby inhibiting the proliferation of keloids, often used after **s**urgical excision to reduce recurrence of keloids.^[27,28]^ Asiaticoside, an active component of Centella asiatica.^[29]^ In vitro experiments showed that asiaticoside can inhibit the growth of keloid fibroblasts and the expression of CTGF in a dose-dependent manner,^[30]^ and play a role in anti-scarring hyperplasia. The combination of multiple drugs inhibits the hyperplasia of keloids in patients after surgery, and alleviates the pain, itching and other problems caused by the hyperplasia from keloid.

It is worth noting that the treatment course of multistage surgery will be longer and patients need to have good compliance. Otherwise, the treatment may fail. Therefore, it is necessary to introduce the surgical procedure to patients before the operation and obtain the cooperation from them.

## 4 Conclusion

We have successfully cured the giant keloid in the chest of the patient by the use of combined multistage surgery with other methods. This has proved beneficial in the treatment for giant keloids, which is worthy of clinical reference.

## Author contributions

**Investigation:** Qingwu Liu, Ping Li, Dingquan Yang.

**Validation:** Dingquan Yang.

**Writing – original draft:** Qingwu Liu, Ping Li, Dingquan Yang.

**Writing – review & editing:** Ping Li, Zhishan Yang, Baoquan Qu, Chunfang Qin, Shengnan Meng, Huijuan Fang, Ruiying Wu, Tiantian Cheng, Dingquan Yang.

Qingwu Liu orcid: 0000-0002-7172-6656.
